# Crystal structure of carbonic anhydrase CaNce103p from the pathogenic yeast *Candida albicans*

**DOI:** 10.1186/s12900-018-0093-4

**Published:** 2018-10-26

**Authors:** Jiří Dostál, Jiří Brynda, Jan Blaha, Stanislav Macháček, Olga Heidingsfeld, Iva Pichová

**Affiliations:** 1 Institute of Organic Chemistry and Biochemistry of the Czech Academy of Sciences, Flemingovo náměstí 2, 166 10 Prague, Czech Republic; 2 Department of Biological and Biochemical Sciences, Faculty of Chemical Technology, University of Pardubice, Studentská 95, 532 10 Pardubice, Czech Republic

**Keywords:** Carbonic anhydrase, *Candida albicans*, Crystal structure, CaNce103p, Substrate tunnel

## Abstract

**Background:**

The pathogenic yeast *Candida albicans* can proliferate in environments with different carbon dioxide concentrations thanks to the carbonic anhydrase CaNce103p, which accelerates spontaneous conversion of carbon dioxide to bicarbonate and vice versa. Without functional CaNce103p, *C. albicans* cannot survive in atmospheric air. CaNce103p falls into the β-carbonic anhydrase class, along with its ortholog ScNce103p from *Saccharomyces cerevisiae*. The crystal structure of CaNce103p is of interest because this enzyme is a potential target for surface disinfectants.

**Results:**

Recombinant CaNce103p was prepared in *E. coli*, and its crystal structure was determined at 2.2 Å resolution. CaNce103p forms a homotetramer organized as a dimer of dimers, in which the dimerization and tetramerization surfaces are perpendicular. Although the physiological role of CaNce103p is similar to that of ScNce103p from baker’s yeast, on the structural level it more closely resembles carbonic anhydrase from the saprophytic fungus *Sordaria macrospora*, which is also tetrameric. Dimerization is mediated by two helices in the N-terminal domain of the subunits. The N-terminus of CaNce103p is flexible**,** and crystals were obtained only upon truncation of the first 29 amino acids. Analysis of CaNce103p variants truncated by 29, 48 and 61 amino acids showed that residues 30–48 are essential for dimerization. Each subunit contains a zinc atom in the active site and displays features characteristic of type I β-carbonic anhydrases. Zinc is tetrahedrally coordinated by one histidine residue, two cysteine residues and a molecule of β-mercaptoethanol originating from the crystallization buffer. The active sites are accessible via substrate tunnels, which are slightly longer and narrower than those observed in other fungal carbonic anhydrases.

**Conclusions:**

CaNce103p is a β-class homotetrameric metalloenzyme composed of two homodimers. Its structure closely resembles those of other β-type carbonic anhydrases, in particular CAS1 from *Sordaria macrospora*. The main differences occur in the N-terminal part and the substrate tunnel. Detailed knowledge of the CaNce103p structure and the properties of the substrate tunnel in particular will facilitate design of selective inhibitors of this enzyme.

## Background

Carbonic anhydrases (CAs) catalyze the interconversion of carbon dioxide and bicarbonate. While the reaction CO_2_ + H_2_O ↔ HCO_3_^−^ + H^+^ proceeds spontaneously, tight control of this process is so important that CAs evolved in all kingdoms of life and arose independently several times during evolution. CAs are a group of structurally unrelated enzymes that share the same catalytic mechanism and can accelerate the spontaneous reaction up to 10,000-fold. Thus, CAs rank among the most efficient enzymes known, with catalytic efficiency approaching the diffusion-control limit [1, 2]. All CAs are metalloenzymes, most often containing zinc coordinated in a tetrahedral geometry by three amino acid residues and a hydroxyl anion. The catalytic mechanism relies on a metal hydroxide derivative formed by water and the metal ion that acts as a strong nucleophile. This nucleophilic species attacks the CO_2_ molecule bound in the active site cavity [3, 4].

Based on sequence and structural features, CAs can be divided into six categories: α, β, γ, δ, ζ and η [4]. The best-studied group, the α-class CAs, were first discovered in vertebrate erythrocytes and later found in prokaryotes, protozoa and plants [4, 5]. β-CAs were first discovered in red clover and fern chloroplasts [6], and their presence has been reported in all other types of organisms except for mammals. Both α and β-CAs have been found in fungi, and all yeast CAs characterized to date belong to the β-class [3].

β-CAs are active as homodimers or higher oligomers formed by these homodimers, with one zinc atom per monomeric subunit [4, 7]. Of the four yeast CAs that have been structurally characterized, two—those from *Saccharomyces cerevisiae* and *Cryptococcus neoformans*—have a dimeric structure. Structures of two CA isoenzymes from *Sordaria macrospora* display tetrameric organization [8].

*Candida albicans*, the most common human fungal pathogen, possesses at least one gene encoding a CA [9]. This gene has been denominated *NCE103* because of its homology to *S. cerevisiae* gene *NCE103* (non-classical export). *ScNCE103* was first identified as a coding sequence for a protein detected in the extracellular space, although it lacks the classical signal peptide [10]. The protein product, ScNce103p, was later found to have CA activity and is particularly important for yeast cells in environments with low CO_2_ concentrations [11, 12].

Compared with non-pathogenic yeasts, *C. albicans* can proliferate in a more diverse range of environments. It can thrive on host skin, mucosa or blood, and it can survive on abiotic surfaces [13]. These sites differ in composition, accessibility of nutrients, pH, and concentration of gases. The CO_2_ concentration in human blood is 150-fold higher than in atmospheric air, and *C. albicans* can adapt to both. CA is crucial for this adaptation. *C. albicans* lacking both *NCE103* alleles is not viable in atmospheric air. Conversely, in the presence of 5.5% CO_2_, *NCE103* expression was nearly undetectable [9, 14].

The indispensability of Nce103p, along with the fact that it is structurally unrelated to mammalian CAs, makes it a promising target for the design of novel antimycotics. Nce103p inhibitors would not act against systemic mycoses because blood contains at least 5% CO_2_, which strongly downregulates *NCE103*. Nevertheless, such inhibitors could serve as components of ointments for topical treatment or surface disinfectants, which may be of particular use in a hospital environment. Nosocomially transmitted infections by *Candida* species represent a serious public health problem, and *C. albicans* infections are associated with high mortality rates, particularly in immunocompromised patients. The structure of CaNce103p from *C. albicans* presented here may facilitate the design and development of such antifungal compounds.

## Results

### Cloning, expression, purification and activity detection of CaNce103p and its truncated versions

To isolate a sufficient amount of CaNce103p for structural studies and facilitate preparation of CaNce103p variants, we developed a recombinant expression system in *E. coli*. The vector pET22b-wtNCE103 was constructed by inserting the CaNce103p coding sequence into a pET22b vector from which the pelB signal sequence for periplasmic localization had been removed. The stop codon at the end of the coding sequence was preserved to prevent attachment of a His-tag to the resulting protein. Four histidine residues naturally occurring at the C-terminus of CaNce103p were sufficient to enable purification using affinity chromatography on a column charged with Ni^2+^ cations.

**Fig. 1:**
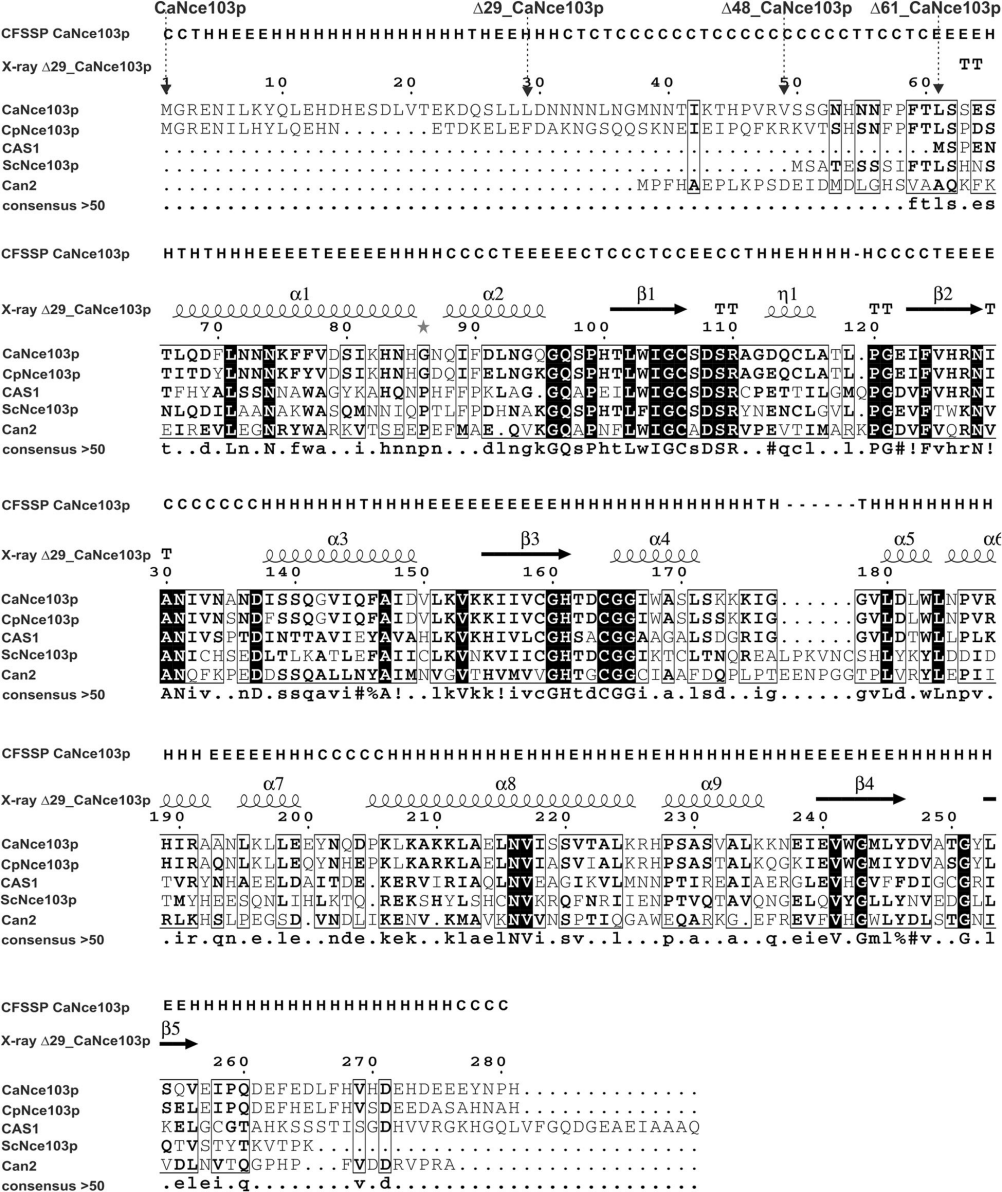
Multiple alignment of fungal β-CAs and secondary structure prediction of CaNce103p. Secondary structure prediction of CaNce103p and sequence multialignment of CaNce103p and CAs from *Candida parapsilosis*, (CpNce103p, CGDID: CAL0000147197), *Saccharomyces cerevisiae* (ScNce103, Swiss-Prot: P53615), *Sordaria macrospora* (CAS1, Swiss-Prot: C1L335), *Cryptococcus neoformans* (Can2, EMBL: Q314V7). The predicted secondary structure regions are indicated as follows: H-Helix, E-Sheet, T-Turn, C-Coil. Black and gray backgrounds denote identical and similar amino acid residues, respectively. Secondary structural elements (X-ray CaNce103p) found in ∆29_CaNce103p are shown as helices and arrows (strands). The black arrows indicate sites of truncation. All sequences were obtained from NCBI databases. Prediction of secondary structure and multialignment was performed using the programs CFSSP [16], MultAlin [28] and ESPript [29]

Truncated versions of CaNce103p were prepared to facilitate crystallization. ScNce103p, the homologous CA from *S. cerevisiae*, was successfully crystallized only upon removal of the first 13 amino acids, which are not conserved among β-CAs [15]. The non-conserved N-terminal part of CaNce103p is even longer than that of ScNce103p (Fig. 1). The vectors pET22b-∆29_CaNCE103, pET22b-∆48_CaNCE103 and pET22b-∆61_CaNCE103 were prepared to express *C. albicans* CA lacking the first 29, 48 and 61 amino acids, respectively.

Truncation by 29 and 61 amino acids was based on secondary structure prediction performed using the program CFSSP [17] (Fig. 1). Deletion of the first 29 residues removes the entire helix predicted to occur at the N-terminus. Deletion of 61 residues removes all the residues before the beginning of the next predicted helix (Fig. 1). Deletion of the first 48 amino acids was motivated by multiple sequence alignment of CAs. The N-terminal part of an orthologous enzyme from the pathogenic yeast *Candida parapsilosis* contains a KR motif, which is a potential target for subtilisin-like processing proteinases. Deleting the first 48 amino acids of CaNce103p removes the homologous position, although CaNce103p does not have a KR motif in its N-terminal part.

**Fig. 2:**
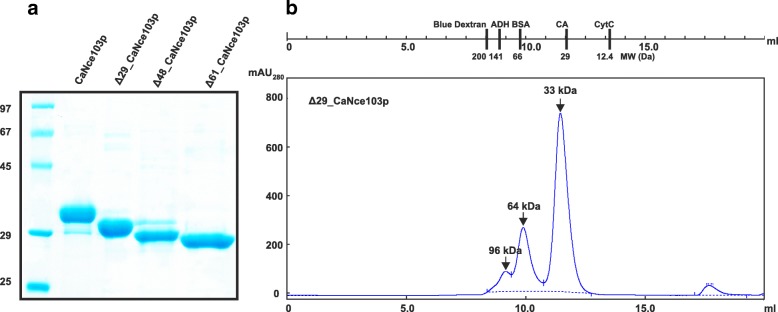
Purification of wt and truncated CaNce103p variants and detection of the oligomeric state of Δ29_CaNce103p. **(a)** 12% SDS-PAGE analysis of CaNce103p, ∆29_CaNce103p, ∆48_CaNce103p and ∆61_CaNce103p (**b**) Elution profile (A_280_) of Δ29_CaNce103p on a Superdex 200 Increase 10/300 GL. A 100 μl aliquot of a 1.0 mg/ml solution of Δ29_CaNce103p was injected. The column was equilibrated and run as described in the Methods section. Molecular weights of monomeric, dimeric and tetrameric forms were estimated by comparison with protein standards (Sigma Aldrich). The upper scale indicates elution volumes for Mw standards from left to right: blue dextran (2000 kDa), alcohol dehydrogenase (ADH, 141 kDa), bovine serum albumin (BSA, 66 kDa), α-carbonic anhydrase from bovine erythrocytes (CA, 29 kDa) and cytochrome C (CytC 12.4 kDa). Arrows with calculated molecular weights indicate elution volumes for Δ29_CaNce103p monomers, dimers and tetramers

To obtain maximal protein yield, we optimized culture conditions including temperature (20–37 °C), cultivation time post-induction (4–20 h) and IPTG concentration (0.2–1.0 mM). The highest yields of soluble CaNce103p, ∆29_CaNce103p, ∆48_CaNce103p and ∆61_CaNce103p were obtained 24 h post-induction with 0.4 mM IPTG with cultivation at 20 °C. The presence of the disulfide bond-reducing reagent β-mercaptoethanol was necessary to keep the protein in a soluble state during the purification process. All CaNce103p variants were purified (Fig. 2a), with average yields of 12–23 mg purified protein per liter of culture for CaNce103p, ∆29_CaNce103p and ∆48_CaNce103p and 2–8 mg/L for ∆61_CaNce103p.

**Fig. 3:**
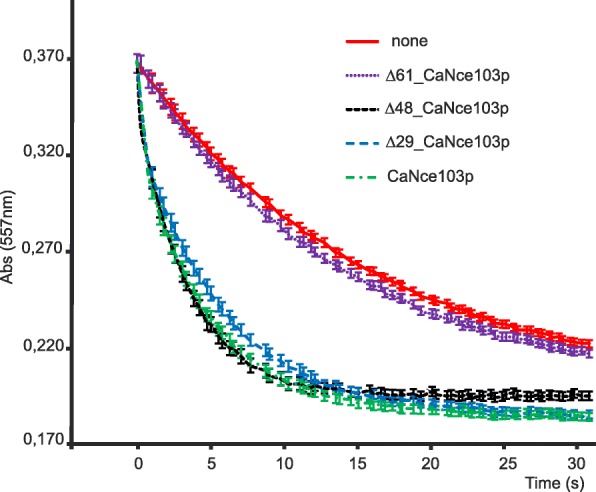
Activity assays of full-lengths and truncated versions of CaNce103p. Hydration of CO_2_ catalyzed by carbonic anhydrase was measured as described in the Methods. The rates of reactions catalyzed by full-length CaNce103p and its truncated versions Δ61_CaNce103p, Δ48_CaNce103p, Δ29_CaNce103p were compared to the rate of spontaneous CO_2_ hydration without enzyme addition

The activity of full-length and truncated versions of CaNce103p detected using the stop-flow pH/dye indicator method [15] (Fig. 3) indicated that truncation by 29 and 48 amino acids did not cause differences in the enzyme activity in comparison with the wild-type full-length CaNce103p. Truncation by 61 amino acids rendered the enzyme inactive. The reaction in presence of Δ61_CaNce103p has a similar rate as the spontaneous hydration of CO_2_. The activity detection also confirmed that β-Mercaptoethanol does not negatively influence the enzyme activity.

### Assessment of the oligomeric structure of Δ29_CaNce103p

Structures of fungal CAs solved to date indicate that these oligomeric enzymes are composed of an even number of identical subunits. We determined the oligomeric state of CaNce103p variants using size-exclusion chromatography. ∆29_CaNce103p formed tetramers (Fig. 3b), which was the highest-order oligomer observed in this study. ∆29_CaNce103p was the only version present as a monomer, dimer and tetramer under our experimental conditions. WT_CaNce103p occurred only as a dimer and tetramer. ∆48_CaNce103p occurred only as a monomer and a precipitate; ∆61_CaNce103p was present only as a precipitate. These findings suggest the importance of the N-terminus for folding and oligomerization of CaNce103p.

### Protein crystallization

Protein crystallization was facilitated by removal of the first 29 amino acids. Our attempts to crystallize full-length CaNce103p or the variants truncated by 48 or 61 residues were unsuccessful. WT_CaNce103p, ∆48_CaNce103p and ∆61_CaNce103p formed precipitates or very small crystals with skin on the drop. Repeated unsuccessful attempts in a variety of crystallization conditions resulted in our decision to focus on ∆29_ CaNce103p. Purified ∆29_ CaNce103p was enzymatically active and crystallized in the form of needles belonging to the Space Group *P*2_1_2_1_2_1_ (Tab. 2), which allowed determination of the structure at 2.2 Å resolution.

### Overall architecture

**Fig. 4:**
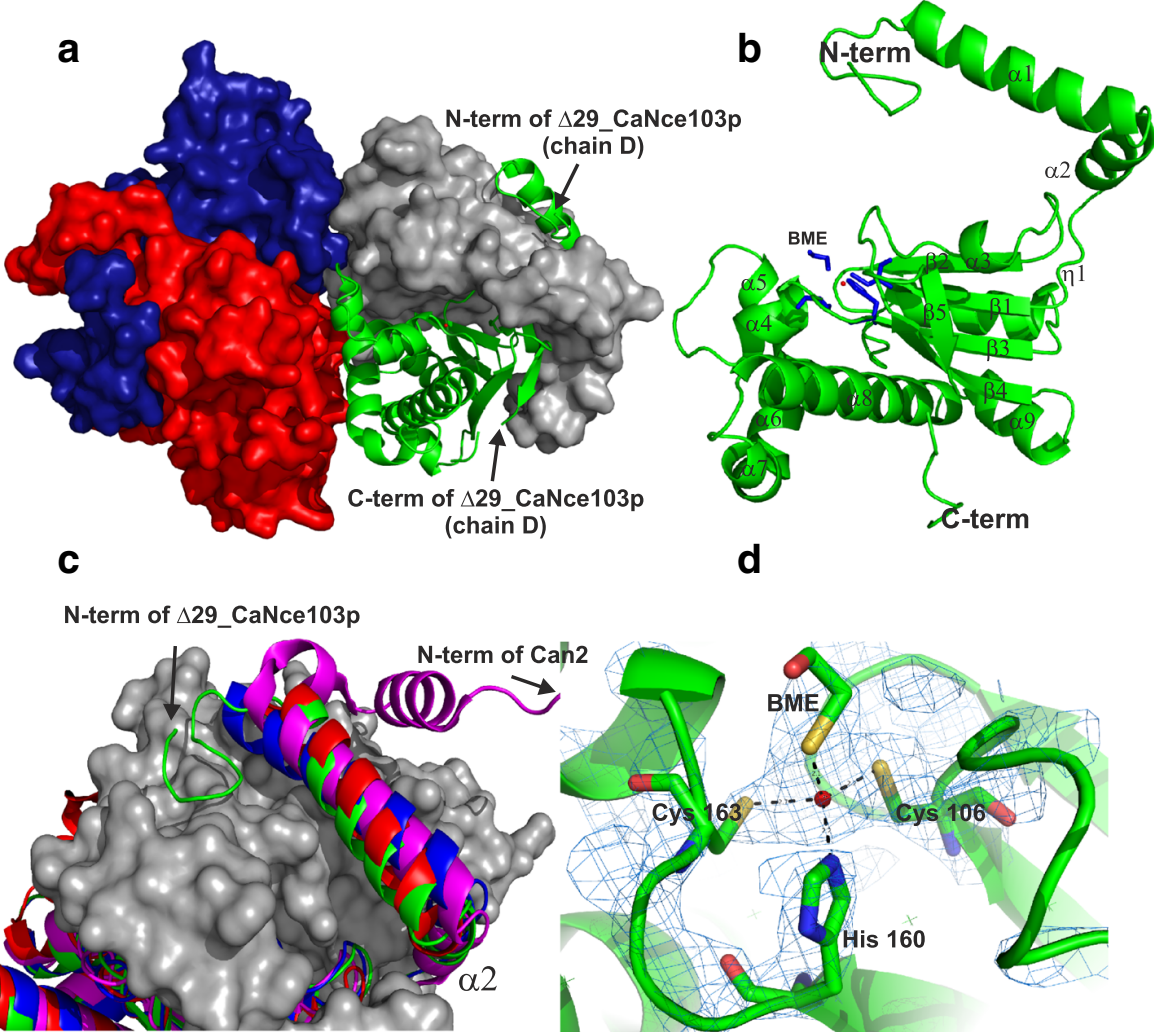
Crystal structures of carbonic anhydrase Δ29_CaNce103p. (**a**) Overall structure of Δ29_CaNce103p illustrating the tetrameric assembly. The tetramer is shown with chains A, B, C (red, blue, cyan) in surface representation and chain D (green) in cartoon representation. (**b**) Δ29_CaNce103p monomer. Secondary structure elements are labeled, and the positions of the N- and C-terminus are indicated. The Zn^2+^ ions are shown as red spheres. Coordinating His160, Cys106, Cys163 and β-mercaptoethanol (blue sticks) are only shown for the D chain. (**c**) Close-up view of superposition of the N-terminal arms of Δ29_CaNce103p (green), ScNce103 (red), CAS1 (blue) and Can2 (magenta). The first two conserved α-helices (α1 and α2) are shown in cartoon representation. The C-terminal subdomain of the second molecule of Δ29_CaNce103p in the asymmetric unit is shown in surface representation (gray). (**d**) Detailed structure of the active site in stick representation. Numbers indicate distances in Å for the contacts, shown as black dashed lines. The 2F_o_-F_c_ electron-density map is contoured at 2.0 σ in blue

The overall structure of CaNce103 is similar to that of CAS1, a β-CA from the plant fungal pathogen *Sordaria macrospora* [8]. It also resembles structures of β-CAs from red algae [17] and bacteria including *Escherichia coli, Vibrio cholerae* and *Haemophilus influenzae* [18–20]. CaNce103 is a complex of four identical subunits organized as a dimer of dimers, in which the dimerization and tetramerization surfaces are mutually perpendicular (Fig. 4a). The subunits in each dimer are interlocked by their N-terminal arms, consisting of two perpendicular helices stretched from the rest of the molecule over the neighboring subunit (Fig. 4b, c). Each monomer provides more than 90 residues to make contact with its dimerization partner, creating an interface of 3458 Å^2^. We calculated the interaction energy stabilizing the dimer to be − 48.8 kcal/mol. Association of two dimers in a tetramer is not as strong; it relies on 33 residues forming an interface of 996 Å^2^. The tetramer is stabilized by an interaction energy of − 11.2 kcal/mol.

The central part of each monomeric subunit is formed by a β-sheet consisting of four parallel strands and one antiparallel strand. This conserved β-structure is flanked on both sides by α-helices. The C-terminal part adjacent to the β-sheet domain is mostly helical. Each monomer contains one zinc atom located in the active site at the bottom of a narrow tunnel, similarly as in ScNce103p [15].

While ∆29_ CaNce103p was the only variant that successfully crystallized, the solved structure corresponds to CaNce103p lacking the first 60 amino acids. This indicates the high flexibility of the N-terminal part of the enzyme.

### CaNce103p active site

The active site of Δ29_CaNce103p is formed by the catalytic Zn^2+^ coordinated by Sγ atom of Cys 106, Nε2 atom of His 160 and Sγ atom of Cys 163 located 2.3 Å from the acceptor atom. According to these data, CaNce103p appears to be a member of the type I β-CAs, the active sites of which are typically formed by two cysteines, one histidine and a fourth ligand—usually water, acetic acid or acetate ion [7]. However, in the present structure, a molecule of β-mercaptoethanol originating from the crystallization buffer fills the fourth position (Fig. 4d).

**Fig. 5:**
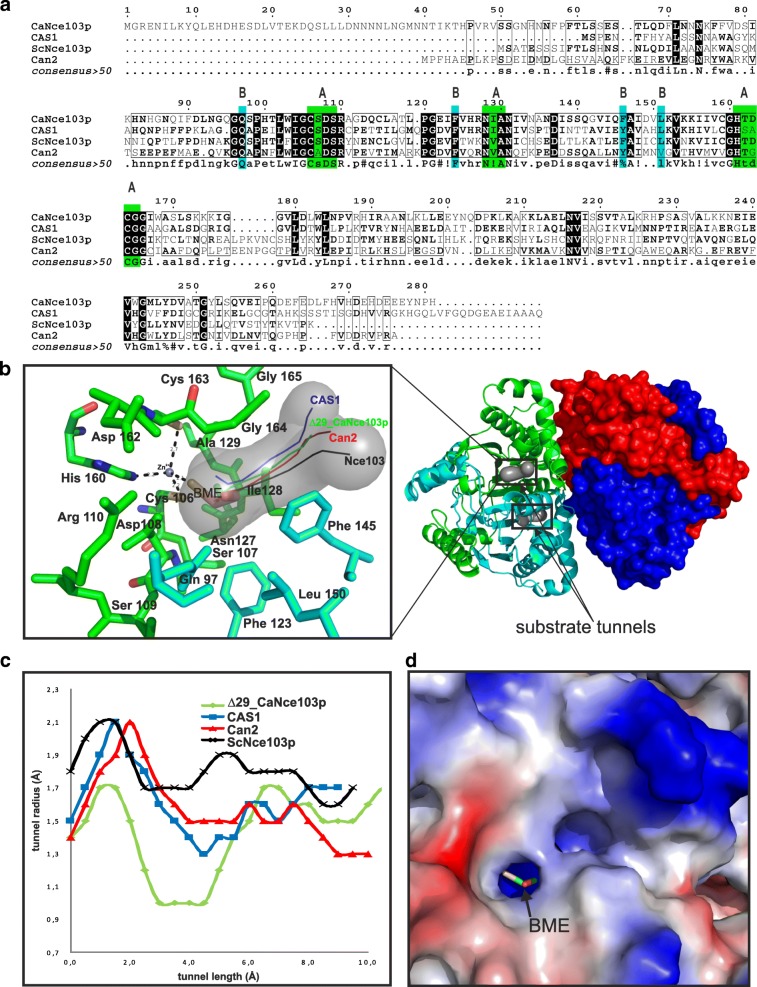
The substrate tunnels of Δ29_ CaNce103p, ScNce103, CAS1 and Can2. **a**) Sequence alignment of Δ29_ CaNce103p, ScNce103, CAS1 and Can2. Active site residues are indicated by asterisks. The residues forming the substrate tunnels are colored as follows: green, subunit A; cyan, subunit B. Multialignment was performed using the program MultiAlin [27]. **b**) Superposition of the simulated substrate tunnels of Δ29_ CaNce103p, ScNce103, CAS1 and Can2. On the left side is a close-up view of the tunnels. On the right is the overall tetrameric structure of Δ29_CaNce103p with the substrate tunnels colored gray. Subunit A is green and subunit B is cyan. The tunnel profiles of each of the CAs are represented by colored lines: green (Δ29_CaNce103p), black (ScNce103), red (Can2) and blue (CAS1). **c**) Line graphs of the substrate tunnel profiles. Simulations of the substrate tunnels were performed using the program CAVER [30]. **d**) Close-up view of the entrance of the Δ29_CaNce103 active site. The image shows the solvent accessible surface colored by electrostatic potential (red for negative, blue for positive)

The zinc coordination sphere is located near the dimer interface. Two of the residues contributing to the zinc coordination sphere are located at the tips of β-sheets (Cys 106 at β1 and His 160 at β3). Cys 163 is located outside of the β-sheet core. The catalytic site is surrounded by amino acids located between the α2 and α4 helices of the contributing monomer units (Figs. 1 and 5a). The contributing residues, most of which are hydrophobic (monomer providing zinc ion ligands: Ile 129, Gly 165; neighboring monomer: Phe 146, Leu 151), create a narrow tunnel (Fig. 4b), which serves as the only point of entry to the positively charged active site (Fig. 5d). The tunnel’s shape and openness may be regulated by the Arg 111 – Asp 163 salt bridge that also contributes to formation of the active site cavity. This salt bridge may function as a pH-dependent regulator of the catalytic activity of Δ29_CaNce103p [21].

### Comparison of CaNce103p to other fungal carbonic anhydrases

We aligned the crystal structure of Δ29_CaNce103p with other known β-CA structures from *Cryptococcus neoformans* (Can2; PDB code: 2W3N), *Saccharomyces cerevisiae* (ScNce103p; PDB code: 3EYX) and *Sordaria macrospora* (CAS1; PDB code: 4O1J). The alignment revealed very high similarity among these homologs. The monomer subunits of all structures are nearly identical, although significant differences occur in the N-terminal part. Of the CAs characterized to date, ScNce103p shares the highest sequence homology with Δ29_CaNce103p (Fig. 1). At the overall structural level, however, Δ29_CaNce103p is more closely related to CAS1, which also forms a tetramer. The root mean square deviation (RMSD) for the superposition of 142 Cα atoms of these proteins is 0.7 Å. CAs from *S. cerevisiae* and *C. neoformans* form dimers, and RMSD values for superposition of ScNce103p and Can2 with CaNce103p are 1.2 Å for 124 Cα pairs and 1.1 Å for 132 Cα pairs, respectively.

The N-terminal part of Δ29_CaNce103p resembles those of CAS1 and ScNce103p, while the Can2 structure includes an additional helix. However, there are similarities in the substrate tunnel region of the Can2 and Δ29_CaNce103p structures. The substrate tunnels have similar shapes and orientations (Fig. 5b), although the middle part of the Δ29_CaNce103p substrate tunnel is rather narrow compared to those of other fungal CAs (Fig. 5c). We observed the most pronounced differences in shape and proportion of the substrate tunnel when comparing the Δ29_CaNce103p and ScNce103p structures, which interestingly share the highest sequence homology. The active site structure and overall structure of Δ29_CaNce103p are nearly identical to those of CAS1.

## Discussion

CAs are being investigated as drug targets and as potential components of carbon sequestration systems to alleviate increasing concentrations of atmospheric CO_2_ [2]. The fungal CA structure presented here is the sixth that has been solved to date, joining structures of CAs from *Aspergillus oryzae*, *Cryptococcus neoformans*, *Saccharomyces cerevisiae* and two CAs from *Sordaria macrospora*. The latter display the highest structural similarity to CaNce103p; one, CAS1, was used as a search model for CaNce103p structure determination. Interestingly, *S. macrospora* is the only fungus investigated to date that can survive in atmospheric air in the absence of a functional CA [8]. In *C. albicans* and *S. cerevisiae*, Nce103p is dispensable only in high CO_2_ concentrations.

Human CAs are structurally distant from yeast CAs. β-CAs do not occur in humans, and therefore may serve as a convenient target for antifungal drugs. However, currently available yeast CA inhibitors lack sufficient selectivity. Characterizing the structure of Nce103p may lead to design of more selective and potent inhibitors, which may have applications as surface disinfectants.

Unlike the orthologous enzyme from baker’s yeast, CaNce103p was found to be a tetramer composed of two dimers. Dimerization is mediated by N-terminal helices from each subunit, formed by amino acids 66–83 and 86–94. N-terminal truncation was necessary to obtain crystals, suggesting that the start of the N-terminal domain is likely flexible and less structured. CaNce103p crystallization required removal of the first 29 residues, while truncation by 13 residues was sufficient for ScNce103p to crystallize.

The segment encompassing residues 30–48 appears to play an important role in multimerization of CaNce103p subunits, as the variant lacking the first 48 amino acids occurred only in monomeric form. However, the conformation of this segment remains unclear because the first 54 amino acids of CaNce103p were not visible in the crystal structure. Interestingly, residues 263–281, which form the end of the CaNce103p C-terminus, also were not visible in our structure. The C-terminal part of CaNce103p is longer than that of the orthologous ScNce103p. Non-conserved, flexible termini might play a role in interactions between CAs and other molecules, calling for further investigation.

The active site of Δ29_CaNce103p does not differ substantially from those of other type I β-CAs. The only distinct feature was the molecule of β-mercaptoethanol coordinating the catalytic zinc ion. β-Mercaptoethanol was an important component of all the buffers used during Δ29_CaNce103p purification, and the presence of a reducing agent appears to be essential for the protein’s stability and solubility. The active site is located at the bottom of the substrate tunnel, the size of which differs from substrate tunnels of other structurally characterized fungal CAs.

Currently available data suggest that CAs from *C. albicans* and *S. cerevisiae* are likely to play a similar physiological role. They are also close homologs at the amino acid sequence level. On the structural level, however, ScNce103p and Δ29_CaNce103p differ.

## Conclusions

In the present work, the crystal structure of N-terminally truncated CA from the pathogenic yeast *Candida albicans* (Δ29_CaNce103p) was determined at 2.2 Å resolution. It is the sixth fungal CA to be structurally characterized by X-ray crystallography. To obtain crystals, truncation of the 29 N-terminal amino acids was necessary. Δ29_CaNce103p forms a homotetramer organized as a dimer of dimers. Although the overall molecular architecture and the active site structure of Δ29_CaNce103p share similarities with other β-class CAs, the N- and C-terminal parts of the monomeric subunits and the substrate tunnel differ. The structure of Δ29_CaNce103p will aid the design of inhibitors that potentially could be incorporated into antimycotic surface disinfectants.

## Methods

### Cloning and expression of CaNce103p

Genomic DNA was isolated from *C. albicans* strain HE109 obtained from the mycological collection of the Faculty of Medicine, Palacky University, Olomouc, Czech Republic. The gene *NCE103* and its truncated versions were amplified using the primers listed in the Table 1 and inserted using the InFusion HD Cloning Kit (Clontech) into a pET22b vector linearized with *Nde*I and *Xho*I restriction enzymes. All DNA segments resulting from PCR were verified by sequencing. The resulting expression vectors were transformed into *Escherichia coli* BL23(DE3). Bacteria were cultivated in Luria-Bertani medium containing 50 μg/ml ampicillin and 0.5 mM ZnSO_4_ in a rotation shaker at 37 °C [8]. When OD_600nm_ reached 0.8, production of CaNce103p was induced by addition of IPTG to a final concentration of 1 mM. The culture was then incubated overnight at 20 °C, and the cells were harvested by centrifugation at 4000 g for 10 min. The cells were resuspended in 10 mM Tris-Cl, pH 8, containing 0.5 M NaCl, and disintegrated using an EmulsiFlex-C3 homogenizer. The cell lysate was centrifuged at 15,000 g for 15 min at 4 °C. CaNce103p was present in the supernatant and was purified using a HiTrap Ni column (GE Healthcare) equilibrated in 10 mM Tris-Cl, pH 8, 0.5 M NaCl. Proteins were eluted with a 0–0.5 M imidazole gradient. The eluate was collected in test tubes containing 20 mM Tris-Cl, pH 8, supplemented with 40 mM β-mercaptoethanol, 400 mM NaCl and 20% glycerol. The ratio of eluate to buffer in the test tubes was 1:1. Selected fractions were pooled and dialyzed against 10 mM Tris-Cl, pH 8, containing 20 mM β-mercaptoethanol, and 10% glycerol. The final purification step was anion-exchange chromatography on a MonoQ column equilibrated in 10 mM Tris-Cl, pH 8, 20 mM β-mercaptoethanol, 10% glycerol. Proteins were eluted using a 0–1 M NaCl gradient. The efficiency of purification was analyzed using SDS-PAGE, Western blotting and activity assays. The amino acid sequences of the obtained proteins were verified by N-terminal protein sequencing and MS mass-fingerprinting analysis.

**Table 1:** Primers used in this study

Construct	Primer name	Sequence	Restriction site
WT	WT_CaNCE103 Nde I F	AAGGAGATATACATATGATGGGTAGAGAAAATATTTTGAA	*Nde*I
	Δ29/Δ48/Δ61_CaNCE103 Xho I R	GGTGGTGGTGCTCGAGTCAATGAGGGTTATATTCTTCTTC	*Xho*I
Δ29	Δ29_CaNCE103 Nde I F	AAGGAGATATACATATGGATAATAATAACAACCTAAACGG	*Nde*I
	Δ29/Δ48/Δ61_CaNCE103 Xho I R	GGTGGTGGTGCTCGAGTCAATGAGGGTTATATTCTTCTTC	*Xho*I
Δ48	Δ48_CaNCE103 Nde I F	AAGGAGATATACATATGGTTAGTTCAGGAAATCATAATAAT	*Nde*I
	Δ29/Δ48/Δ61_CaNCE103 Xho I R	GGTGGTGGTGCTCGAGTCAATGAGGGTTATATTCTTCTTC	*Xho*I
Δ61	Δ61_CaNCE103 Nde I F	AAGGAGATATACATATGTCTTCAGAATCTACATTACAAGATTT	*Nde*I
	Δ29/Δ48/Δ61_CaNCE103 Xho I R	GGTGGTGGTGCTCGAGTCAATGAGGGTTATATTCTTCTTC	*Xho*I

### Enzyme activity

Carbonic anhydrase activity was tested using a colorimetric assay to measure CO_2_ hydration. Samples of purified proteins were added to final concentration of 0.005 mg/ml to 5 mM HEPES, pH 7.5, containing 20 mM Na_2_SO_4_ and 200 μM phenol red. This suspension was mixed in a 1:1 volume ratio with 3.2 mM CO_2_ solution using a stopped flow device (Rapid Mix accessory RX2000 from Applied Photophysics), and the reaction was monitored using a spectrophotometer (Unicam UV-510 from Thermo Spectronic). Absorbance at 557 nm was recorded for 30 s but only the first second was used for the activity analysis. Control reactions were performed in the absence of enzyme.

### Size exclusion chromatography

To determine the oligomeric state of carbonic anhydrases, size exclusion chromatography was performed using an HR200 Increase column equilibrated in 10 mM Tris-Cl, pH 8, 20 mM β-mercaptoethanol. The flow rate was adjusted to 0.5 ml/min, and the column was calibrated using the Gel Filtration Markers Kit for molecular weights 12,000–200,000 from Sigma Aldrich.

### Crystallization, data collection and structure determination

CaNce103p and its truncated versions (Δ29_CaNce103p, Δ48_CaNce103p, Δ61_CaNce103p) were concentrated using an Amicon Ultra-30 ultrafiltration device (Millipore) to 10 mg/ml. Initial crystallization trials were performed with the help of a Gryphon crystallization workstation (Art Robbins Instruments) by the sitting drop vapor diffusion method at 18 °C in 96-well plates; 0.2 μl protein solution was mixed with 0.2 μl well solution and the mixture was equilibrated over a 200 μl reservoir solution. PEGs Suite I and JCSG Core I Suite (QIAGEN) were used for the crystallization condition screen. Initial microcrystals of Δ29_CaNce103p appeared in several days under the following conditions: 0.2 M ammonium acetate or 0.2 M magnesium chloride, 0.1 M Bis-Tris, pH 5.5, 25% (*w*/*v*) PEG 3350 or 45% (w/v) MPD. Further subsequent optimization of crystallization conditions involved changing to the hanging drop mode, which was performed in NeXtal plates (Qiagen) for easy crystal manipulation. Final crystals were obtained by mixing 3 μl Δ29_CaNce103p with 1 μl reservoir solution composed of 0.1 M ammonium acetate, pH 5.5, 25% PEG 3350.

For data collection, the crystal was frozen in liquid nitrogen. For cryoprotection, the crystals were soaked for 10 s in the corresponding reservoir solution supplemented with 25% (*v*/v) glycerol. Diffraction data for Δ29_CaNce103p were collected to 2.2 Å resolution at 100 K using the MX14.2 beamline at BESSY, Berlin, Germany [22]. Diffraction data were processed using the XDS suite [23, 24] using XDSAPP2.0 [25].

The structure was determined by molecular replacement using the program Molrep. β-CA from *Sordaria macrospora* (PDB ID: 4O1J) was used as the search model. Model refinement was carried out using the program Phenix.refine [26] from the Phenix package (version 1.9–1692) [27], and the final cycles were performed with REFMAC 5.2 from the CCP4 package. Manual building was performed using Coot. The quality of the final model was validated with the Molprobity server.

Crystal parameters, data collection and refinement statistics are summarized in Table 2. Structural representations were prepared with the program PyMOL. Atomic coordinates and experimental structure factors have been deposited in the Protein Data Bank under code 6GWU.

**Table 2:** Crystal data and diffraction data collection and refinement statistics

Data-collection statistics	
Wavelength (Å)	0.9184
Space group	*P2*_*1*_ *2*_*1*_ *2*_*1*_
Unit-cell parameters (Å, °)	a = 69.37, b = 90.29, c = 167.12, α = 90.0, β = 90.0, γ = 90.0
No. of molecules in asymmetric unit	4
Resolution range (Å)	50–2.2 (2.33–2.20)
No. of unique reflections	53,038 (8439)
Multiplicity	4.5 (4.6)
Completeness (%)	98.1 (98.2)
Rmerge†	16.5 (238.5)
Average I/σ(I)	8.27 (0.62)
Wilson B (Å^2^)	49.0
Refinement statistics	
Resolution range (Å)	45.95–2.20 (2.3–2.20)
No. of reflections in working set	51,601 (6327)
No. of reflections in test set	1053 (129)
Rwork‡ (%)	24.1 (40.5)
Rfree§ (%)	27.9 (43.0)
Rall (%)	24.1
R.m.s.d., bond lengths (Å)	0.019
R.m.s.d., bond angles (Å)	1.57
No. of non-H atoms in asymmetric unit	6269
No. of water molecules in asymmetric unit	35
Mean ADP (Å^2^)	81.6
Main chain (A/B/C/D)	76.5/75.8/92.0/82.2
Side chain (A/B/C/D)	80.8/80.3/93.7/86.4
Water	62.3
Residues in alternative conformations	0
Ramachandran plot statistics	
Residues in favoured regions (%)	95.7
Residues in allowed regions (%)	4.1

## Abbreviations

Can2: Carbonic anhydrase from *Cryptococcus neoformans*
CaNce103p: Carbonic anhydrase from *Candida albicans*
CAs: Carbonic anhydrases
CAS1: Carbonic anhydrase from *Sordaria macrospora*
*NCE*: Non-classical export
RMSD: Root mean square deviation
ScNce103p: Carbonic anhydrase from *Saccharomyces cerevisiae*
